# Prognostic influences of BCL1 and BCL2 expression on disease-free survival in breast cancer

**DOI:** 10.1038/s41598-021-90506-x

**Published:** 2021-06-07

**Authors:** Ki-Tae Hwang, Young A. Kim, Jongjin Kim, Hyeon Jeong Oh, Jeong Hwan Park, In Sil Choi, Jin Hyun Park, Sohee Oh, Ajung Chu, Jong Yoon Lee, Kyu Ri Hwang

**Affiliations:** 1 Department of Surgery, Seoul Metropolitan Government Seoul National University Boramae Medical Center, 39, Boramae-Gil, Dongjak-gu, Seoul, 156-707 Republic of Korea; 2 Department of Pathology, Seoul Metropolitan Government Seoul National University Boramae Medical Center, Seoul, Republic of Korea; 3 Department of Pathology, National Cancer Center, Goyang-si, Gyeonggi-do Republic of Korea; 4 Department of Internal Medicine, Seoul Metropolitan Government Seoul National University Boramae Medical Center, Seoul, Republic of Korea; 5 Medical Research Collaborating Center, Seoul Metropolitan Government Seoul National University Boramae Medical Center, Seoul, Republic of Korea; 6 Department of Radiology, Seoul Metropolitan Government Seoul National University Boramae Medical Center, Seoul, Republic of Korea; 7 Department of Obstetrics and Gynecology, Seoul Metropolitan Government Seoul National University Boramae Medical Center, Seoul, Republic of Korea

**Keywords:** Cancer, Breast cancer

## Abstract

We investigated the prognostic influences of BCL1 and BCL2 expression on disease-free survival in breast cancer patients. BCL1 and BCL2 expression statuses were assessed by immunohistochemistry using tissue microarrays from 393 breast cancer patients. The Kaplan–Meier estimator and log-rank test were used for survival analyses. The Cox proportional hazards model was used to calculate hazard ratio (HR) and the 95% confidence interval (CI) of survival analyses. BCL1 expression revealed no impact on survival. The high BCL2 group showed superior disease-free survival compared with the low BCL2 group (*p* = 0.002), especially regarding local recurrence-free survival (*p* = 0.045) and systemic recurrence-free survival (*p* = 0.002). BCL2 expression was a significant prognostic factor by univariable analysis (HR, 0.528; 95% CI, 0.353–0.790; *p* = 0.002) and by multivariable analysis (HR, 0.547; 95% CI, 0.362–0.826; *p* = 0.004). High BCL2 expression was associated with higher disease-free survival in the hormone receptor (HRc)-positive and human epidermal growth factor receptor 2 (HER2)-negative (HRc(+)/HER2(−)) subtype only (*p* = 0.002). The high BCL2 group was associated with positive estrogen receptor (ER), positive progesterone receptor (PR), low histologic grade, and age ≤ 50 years. BCL1 expression had no prognostic impact, but BCL2 expression was a significant independent prognostic factor. High BCL2 expression was associated with higher disease-free survival, especially regarding local recurrence and systemic recurrence. The prognostic effect of BCL2 expression was effective only in the HRc(+)/HER2(−) subtype. Favorable clinicopathologic features and a strong association with the ER/PR status could partly explain the superior prognosis of the high BCL2 group. BCL2 expression could be utilized to assess the prognosis of breast cancer patients in clinical settings.

## Introduction

Both BCL1 and BCL2 are known to have oncogenic activities in various human malignancies and are key proteins in the induction of cellular proliferation and cancer progression^1–6^. Previous studies have investigated the prognostic roles of BCL1 and BCL2 in various human cancers including breast cancer. Although BCL1 and BCL2 were expected to be adverse prognostic indicators in breast cancer, the results have been largely inconsistent.

*BCL1* is reported to be a major oncogene in various human solid cancers^1–4^. *BCL1* is located on chromosome 11q13.3 and is also known as *CCND1*, *cyclin D1*, and *PRAD1*. The BCL1 protein, otherwise known as cyclin D1, is one of the D-type cyclins and is a crucial protein for cell cycle regulation, especially in the process of the G0/S transition. BCL1 overexpression, which is frequently observed in human malignancies, induces shortening of the G0/S transition time, activating rapid cell growth and neoplastic growth^1–4^.

*BCL2* is also regarded to be an oncogene in various malignancies, especially in leukemia and lymphoma, and is a key factor for the regulation of cellular apoptosis. *BCL2* is located on chromosome 18q21 and encodes an integral outer mitochondrial membrane protein that blocks the apoptotic death of cells. BCL2 overexpression inhibits apoptotic cell death and activates cellular proliferation and tumor progression^5,6^. BCL1 and BCL2 also reportedly interact: BCL2 overexpression induces BCL1 expression in human breast epithelial cells^7^.

Although both BCL1 and BCL2 are predicted to be associated with an adverse prognosis in breast cancer, previous studies have reported inconsistent results. Some studies reported the adverse effect of BCL1 on breast cancer prognosis^8–10^, whereas others reported no association between BCL1 expression and survival^11,12^. Furthermore, a favorable prognostic impact of BCL1 was also described^13–15^. BCL2 is also believed to have an adverse influence on breast cancer survival, but most previous studies have reported a favorable prognostic effect^16–20^. However, some studies reported no association between BCL2 expression and survival^21^, and several studies reported that BCL2 is an adverse prognostic indicator^22^.

This study investigates the prognostic effects of BCL1 and BCL2 expression in breast cancer by analyzing tissue microarray data of primary breast cancers. The association of BCL1 and BCL2 expression with various clinicopathologic features as well as different breast cancer subtypes was also investigated.

## Methods

### Study subjects

Primary invasive breast cancer patients have been prospectively registered in the Boramae Hospital Breast Cancer Registry. In 2012, we made tissue microarrays using cancer tissues from 420 registered patients^23^. Among these, 13 patients were excluded due to insufficient information from immunostaining results. Three patients were further excluded because of duplication. An additional 11 patients, who were initially diagnosed as stage IV, were also excluded. Therefore, 393 operable primary breast cancer patients were enrolled in this study. This study was approved by the Institutional Review Boards of the Seoul Metropolitan Government Seoul National University Boramae Medical Center (approval number: 16-2016-82). All methods including data acquisition and analysis were performed in compliance with protocols approved by the Institutional Review Boards. Written informed consent was obtained from all participants prior to the study.

### Preparation of tissue microarrays and immunohistochemistry of BCL1 and BCL2

Tissue microarrays were constructed with 2 mm diameter cores of representative tumor areas from formalin-fixed paraffin-embedded tissue blocks. Immunohistochemical staining was performed using rabbit anti-Cyclin D1 (SP4-R) monoclonal antibody (ready-to-use format, Ventana-Roche Diagnostics, Indianapolis, IN) for BCL1 and mouse anti-BCL2 (Clone 124) monoclonal antibody (1:200, DAKO-Agilent Technologies, Santa Clara, CA) for BCL2.

BCL1 staining was not detected in normal breast cancer tissues (Fig. S1A). BCL1 showed heterogeneous nuclear staining in cases with positive staining of BCL1 in breast cancer tissues (Fig. S1C). BCL2 staining was detected in normal breast cancer tissues (Fig. S1D). BCL2 showed homogenous cytoplasmic staining both in normal breast tissues (Fig. S1D) and breast cancer tissues with positive BCL2 staining (Fig. S1F). BCL2 staining was detected in terminal duct lobular units and reactive lymphocytes but not in the stromal cells of normal or cancer tissues (Fig. S1D, F). Staining results were evaluated based on the proportion and average intensity of nuclear staining for BCL1 and cytoplasmic staining for BCL2 in stained tumor cells. Proportion scores were dichotomously classified as follows: ≤ 10% of tumor cells vs. > 10% of tumor cells. Intensity scores were scored as follows: 0, negative; 1, weak; 2, moderate; 3, strong (Figs. S2 and S3). The Allred score was calculated as the sum of the proportion score and the intensity score, according to previous reports^24,25^.

### Defining low vs. high expression of BCL1 and BCL2

Significant survival differences for BCL1 were not observed at any cut-off values of the Allred scores (Fig. S4). In this study, high BCL1 expression was designated when the Allred score was greater than 6 because the distribution of subject numbers into two groups was most even under this condition. For BCL2, the high BCL2 group showed significantly superior survival compared with the low BCL2 group when cut-off values for the Allred scores were set at 2 (*p* = 0.002), 3 (*p* = 0.001), and 4 (*p* = 0.009) (Fig. S5). In this study, high BCL2 expression was defined when the Allred score was greater than 2 because the distribution of subject numbers was most even under this criterion. Significant survival differences were not observed with regards to either the intensity score or the proportion score in terms of BCL1. The high BCL2 group revealed better survival with regards to both the BCL2 intensity (*p* = 0.002; intensity score 1, 2, 3 vs. 0) score and proportion score (*p* = 0.002; proportion score > 10% vs. ≤ 10%) (Fig. S6).

### Definition of variables

The stage of the cancer was described according to the 8th edition of the American Joint Committee on Cancer. The status of the estrogen receptor (ER) and the progesterone receptor (PR) was defined based on the result of the immunohistochemical test^25,26^. The human epidermal growth factor receptor 2 (HER2) status was defined as positive or negative based on the immunohistochemical results and the in situ hybridization test^27^. The hormone receptor (HRc) status was defined as positive when the immunohistochemical test for either ER or PR was positive. Breast cancer subtypes were classified into four groups according to HRc and HER2: HRc(+)/HER2(−), HRc(+)/HER2(+), HRc(−)/HER2(+), and HRc(−)/HER2(−). Histologic grade followed the modified Scarff-Bloom-Richardson grading system. Body mass index was defined as the ratio of body weight in kilograms to height in square meters.

### Statistical analyses

A two-sample *t*-test was used to determine statistical differences between mean ages, and Pearson’s χ^2^ test was used to determine statistical differences between all other baseline characteristics. Recurrence was defined as any first event of local, regional, systemic, and/or contralateral breast cancer relapse. Time duration for disease-free survival was defined as the time difference from the operation to any recurrence. The Kaplan–Meier estimator was used to analyze survival rates, and a log-rank test was used to determine the significance of differences between survival curves. The Cox proportional hazards model was used to calculate the hazard ratio (HR) and 95% confidence interval (CI). All statistical analyses were carried out using IBM SPSS Statistics, version 26.0 (IBM Corp., Armonk, NY). All tests were two-sided, and statistical significance was defined as when the *p* value was less than 0.05.

## Results

### Baseline characteristics of study subjects

The mean age of the 393 study subjects was 53.4 ± 12.3 years (median, 51.0 years; range, 25–87 years). Operation dates occurred between July 1999 and April 2012. The mean durations for post-operative disease-free survival were 87.5 ± 50.3 months (median, 90.0 months; range, 1–216 months). There were 103 recurrences, and the numbers of local, regional, systemic, and contralateral breast cancer recurrence were 18, 16, 64, and 8, respectively. The numbers of subjects with low and high BCL1 expression were 174 (44.3%) and 219 (55.7%), respectively. The numbers of subjects with low and high BCL2 expression were 205 (52.2%) and 188 (47.8%), respectively (Table 1). The high BCL1 group included higher proportions of patients with ER-positive, PR-positive, and low histologic grade compared with the low BCL1 group. The high BCL2 group showed higher proportions of patients with ER-positive, PR-positive, low histologic grade, and age ≤ 50 years compared with the low BCL2 group. The high BCL1 and high BCL2 groups showed higher proportions of the HRc(+)/HER2(−) and HRc(+)/HER2(+) subtypes and lower proportions of the HRc(−)/HER2(+) and HRc(−)/HER2(−) subtypes. The high BCL1 group received more anti-HER2 therapy and endocrine therapy. The high BCL2 group received more endocrine therapy. High BCL1 expression was associated with high BCL2 expression (*p* < 0.001). Baseline characteristics of study subjects according to BCL1 and BCL2 statuses are summarized in Table 1.

**Table 1:** Baseline characteristics of study subjects according to BCL1 and BCL2 statuses.

Characteristics	All		Low BCL1		High BCL1		*p*^a^	Low BCL2		High BCL2		*p*^a^
	No	%	No	%	No	%		No	%	No	%	
**Total**	393	100.0%	174	44.3%	219	55.7%		205	52.2%	188	47.8%	
**Mean age (years)**	53.4 ± 12.3		54.3 ± 11.7		52.6 ± 12.7		0.163	54.1 ± 12.2		52.6 ± 12.4		0.248
**Tumor size (cm)**							0.980					0.115
≤ 2	133	33.8%	59	33.9%	74	33.8%		62	30.2%	71	37.8%	
> 2	260	66.2%	115	66.1%	145	66.2%		143	69.8%	117	62.2%	
**Nodal positivity**							0.373					0.137
Negative	216	55.0%	100	57.5%	116	53.0%		120	58.5%	96	51.1%	
Positive	177	45.0%	74	42.5%	103	47.0%		85	41.5%	92	48.9%	
**Stage**							0.442					0.845
Stage I	95	24.2%	40	23.0%	55	25.1%		49	23.9%	46	24.5%	
Stage II	203	51.7%	96	55.2%	107	48.9%		104	50.7%	99	52.7%	
Stage III	95	24.2%	38	21.8%	57	26.0%		52	25.4%	43	22.9%	
**Estrogen receptor**							< 0.001					< 0.001
Negative	130	33.1%	92	52.9%	38	17.4%		104	50.7%	26	13.8%	
Positive	263	66.9%	82	47.1%	181	82.6%		101	49.3%	162	86.2%	
**Progesterone receptor**							< 0.001					< 0.001
Negative	151	38.4%	93	53.4%	58	26.5%		106	51.7%	45	23.9%	
Positive	242	61.6%	81	46.6%	161	73.5%		99	48.3%	143	76.1%	
**Hormone receptor**							< 0.001					< 0.001
Negative	102	26.0%	74	42.5%	28	12.8%		80	39.0%	22	11.7%	
Positive	291	74.0%	100	57.5%	191	87.2%		125	61.0%	166	88.3%	
**HER2**							0.922					0.617
Negative	310	78.9%	138	79.3%	172	78.5%		158	77.1%	152	80.9%	
Positive	80	20.4%	35	20.1%	45	20.5%		45	22.0%	35	18.6%	
Unknown	3	0.8%	1	0.6%	2	0.9%		2	1.0%	1	0.5%	
**Subtype**							< 0.001					< 0.001
HRc(+)/HER2(−)	237	60.3%	82	47.1%	155	70.8%		100	48.8%	137	72.9%	
HRc(+)/HER2(+)	52	13.2%	18	10.3%	34	15.5%		24	11.7%	28	14.9%	
HRc(−)/HER2(+)	28	7.1%	17	9.8%	11	5.0%		21	10.2%	7	3.7%	
HRc(−)/HER2(−)	73	18.6%	56	32.2%	17	7.8%		58	28.3%	15	8.0%	
Unknown	3	0.8%	1	0.6%	2	0.9%		2	1.0%	1	0.5%	
**Histologic grade**							0.006					< 0.001
1,2	227	57.8%	85	48.9%	142	64.8%		96	46.8%	131	69.7%	
3	163	41.5%	87	50.0%	76	34.7%		107	52.2%	56	29.8%	
Unknown	3	0.8%	2	1.1%	1	0.5%		2	1.0%	1	0.5%	
**Lymphovascular invasion**							0.435					0.710
Negative	236	60.1%	109	62.6%	127	58.0%		126	61.5%	110	58.5%	
Positive	154	39.2%	63	36.2%	91	41.6%		77	37.6%	77	41.0%	
Unknown	3	0.8%	2	1.1%	1	0.5%		2	1.0%	1	0.5%	
**Age (years)**							0.089					0.026
≤ 50	186	47.3%	74	42.5%	112	51.1%		86	42.0%	100	53.2%	
> 50	207	52.7%	100	57.5%	107	48.9%		119	58.0%	88	46.8%	
**Body mass index (kg/m**^**2**^**)**							0.175					0.100
≤ 25	227	57.8%	99	56.9%	128	58.4%		128	62.4%	99	52.7%	
> 25	162	41.2%	75	43.1%	87	39.7%		76	37.1%	86	45.7%	
Unknown	4	1.0%	0	0.0%	4	1.8%		1	0.5%	3	1.6%	
**Operation**							0.094					0.060
Lumpectomy	114	29.0%	43	24.7%	71	32.4%		51	24.9%	63	33.5%	
Mastectomy	279	71.0%	131	75.3%	148	67.6%		154	75.1%	125	66.5%	
**Radiation therapy**							0.372					0.843
No	208	52.9%	99	56.9%	109	49.8%		106	51.7%	102	54.3%	
Yes	180	45.8%	73	42.0%	107	48.9%		96	46.8%	84	44.7%	
Unknown	5	1.3%	2	1.1%	3	1.4%		3	1.5%	2	1.1%	
**Chemotherapy**							0.344					0.286
No	75	19.1%	30	17.2%	45	20.5%		33	16.1%	42	22.3%	
Yes	314	79.9%	141	81.0%	173	79.0%		170	82.9%	144	76.6%	
Unknown	4	1.0%	3	1.7%	1	0.5%		2	1.0%	2	1.1%	
**Anti-HER2 therapy**							0.033					0.944
No	375	95.4%	169	97.1%	206	94.1%		195	95.1%	180	95.7%	
Yes	16	4.1%	3	1.7%	13	5.9%		9	4.4%	7	3.7%	
Unknown	2	0.5%	2	1.1%	0	0.0%		1	0.5%	1	0.5%	
**Endocrine therapy**							< 0.001					< 0.001
No	104	26.5%	64	36.8%	40	18.3%		82	40.0%	22	11.7%	
Yes	285	72.5%	107	61.5%	178	81.3%		121	59.0%	164	87.2%	
Unknown	4	1.0%	3	1.7%	1	0.5%		2	1.0%	2	1.1%	
**BCL1**												< 0.001
Low	174	44.3%						116	56.6%	58	30.9%	
High	219	55.7%						89	43.4%	130	69.1%	
**BCL2**							< 0.001					
Low	205	52.2%	116	66.7%	89	40.6%						
High	188	47.8%	58	33.3%	130	59.4%						

*HER2* human epidermal growth factor receptor 2, *HRc* hormone receptor.

^a^*P* value for mean age was calculated by *t*-test and all the other *p* values were calculated by *χ*^2^ test.

### Survival analysis according to BCL1 and BCL2 statuses

**Figure 1:**
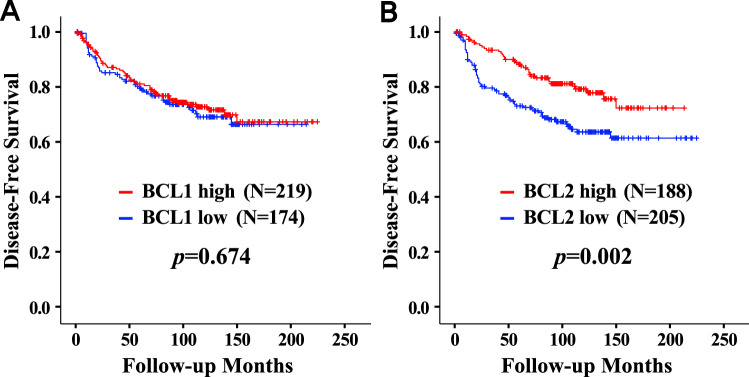
Disease-free survival curves according to BCL1 and BCL2 statuses. Disease-free survival according to BCL1 status (**A**) and BCL2 status (**B**).

**Figure 2:**
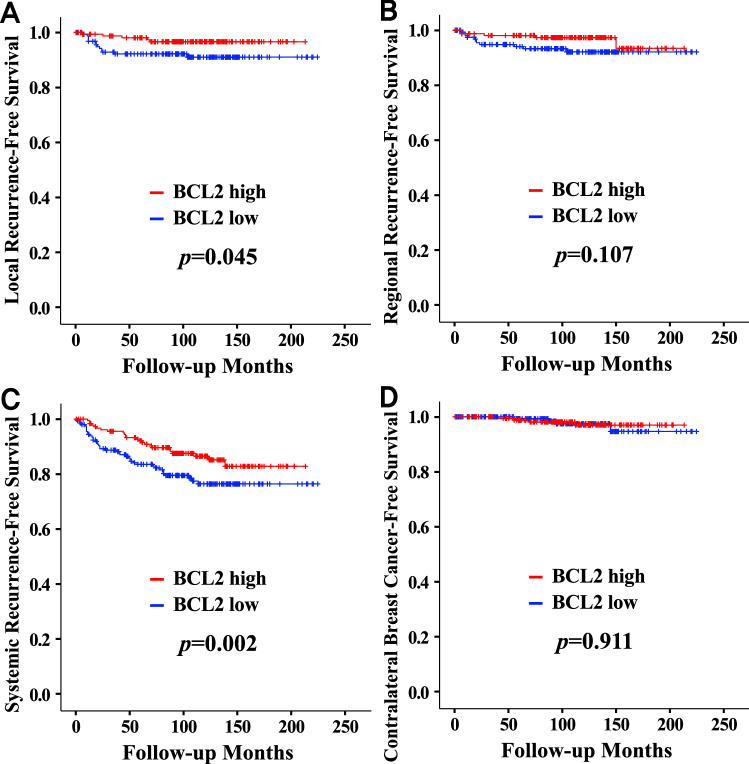
Detailed disease-free survival curves according to BCL2 status. Local recurrence-free survival (**A**), regional recurrence-free survival (**B**), systemic recurrence-free survival (**C**), and contralateral breast cancer recurrence-free survival (**D**).

There was no significant association between BCL1 expression and disease-free survival (Fig. 1A). BCL1 expression revealed no survival differences regarding any recurrence types including local recurrence, regional recurrence, systemic recurrence, and contralateral breast cancer (data not shown). A prognostic effect of BCL1 expression was not observed in unselected breast cancers as well as in HRc-positive or HRc-negative breast cancers (data not shown). However, the high BCL2 group showed superior disease-free survival (*p* = 0.002) compared with the low BCL2 group (Fig. 1B). The high BCL2 group showed superior local recurrence-free survival (*p* = 0.045) and systemic recurrence-free survival (*p* = 0.002). However, BCL2 status did not reveal a significant survival difference in terms of regional recurrence-free survival and contralateral breast cancer-free survival (Fig. 2). The high BCL2 group showed better disease-free survival (*p* = 0.004) in HRc-positive breast cancers but there was no prognostic significance of BCL2 expression in HRc-negative breast cancers (Fig. S7). Detailed survival rates according to BCL2 status for disease-free survival are described in Table S1.

### Univariable and multivariable analyses according to BCL2 status

BCL2 expression was a significant prognostic factor by univariable analysis (HR, 0.528; 95% CI, 0.353–0.790; *p* = 0.002; Table 2). By multivariable analyses, BCL2 expression was also an independent prognostic factor in both model 1 (HR, 0.488; 95% CI, 0.305–0.782; *p* = 0.003) and model 2 (HR, 0.532; 95% CI, 0.353–0.801; *p* = 0.003).

**Table 2:** Univariable and multivariable analyses regarding disease-free survival.

Characteristics (All)	Univariable analysis				Multivariable analysis							
					Model 1^a^				Model 2^b^			
	HR	95% CI		*p*	HR	95% CI		*p*	HR	95% CI		*p*
BCL2, high vs low	0.528	0.353	0.790	0.002	0.488	0.305	0.782	0.003	0.532	0.353	0.801	0.003
Tumor size (cm), > 2 vs ≤ 2	2.268	1.404	3.665	0.001	1.454	0.862	2.452	0.160	1.400	0.854	2.295	0.183
Nodal positivity, positive vs negative	3.141	2.075	4.756	< 0.001	2.279	1.359	3.823	0.002	2.357	1.440	3.859	0.001
Estrogen receptor, positive vs negative	0.895	0.595	1.346	0.593	1.483	0.803	2.740	0.208				
Progesterone receptor, positive vs negative	0.916	0.616	1.361	0.663	0.766	0.455	1.288	0.314				
HER2, positive vs negative	1.108	0.697	1.762	0.665	0.782	0.443	1.380	0.396				
Histologic grade, 3 vs 1,2	1.332	0.904	1.961	0.147	1.222	0.795	1.880	0.360				
Lymphovascular invasion, positive vs negative	2.383	1.605	3.537	< 0.001	1.785	1.105	2.885	0.018	1.569	0.999	2.464	0.051
Age (years), > 50 vs ≤ 50	1.087	0.738	1.601	0.674	0.733	0.467	1.148	0.175				
Body mass index (kg/m^2^), > 25 vs ≤ 25	1.049	0.709	1.552	0.812	1.003	0.657	1.533	0.988				
Operation, mastectomy vs lumpectomy	3.856	2.061	7.211	< 0.001	2.794	1.353	5.773	0.006	2.872	1.520	5.427	0.001
Radiation therapy, yes vs no	0.817	0.551	1.211	0.315	0.898	0.534	1.508	0.683				
Chemotherapy, yes vs no	0.842	0.521	1.363	0.485	0.648	0.372	1.129	0.125				
Anti-HER2 therapy, yes vs no	1.990	0.922	4.297	0.080	2.233	0.877	5.686	0.092				
Endocrine therapy, yes vs no	0.738	0.482	1.130	0.162	0.898	0.463	1.744	0.751				

*CI* confidence interval, *HER2* human epidermal growth factor receptor 2, *HR* hazard ratio.

^a^BCL2 factor was adjusted with all of 14 clinicopathologic factors including tumor size, nodal positivity, estrogen receptor, progesterone receptor, HER2, histologic grade, lymphovascular invasion, age, body mass index, operation, radiation therapy, chemotherapy, anti-HER2 therapy, and endocrine therapy.

^b^BCL2 factor was adjusted with 4 clinicopathologic factors, which were statistically significant by univariable analysis, including tumor size, nodal positivity, lymphovascular invasion, and operation.

### Subgroup survival analyses according to BCL2 status

**Figure 3:**
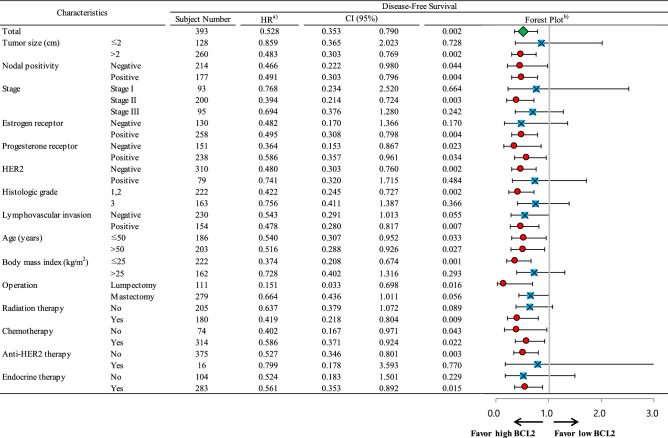

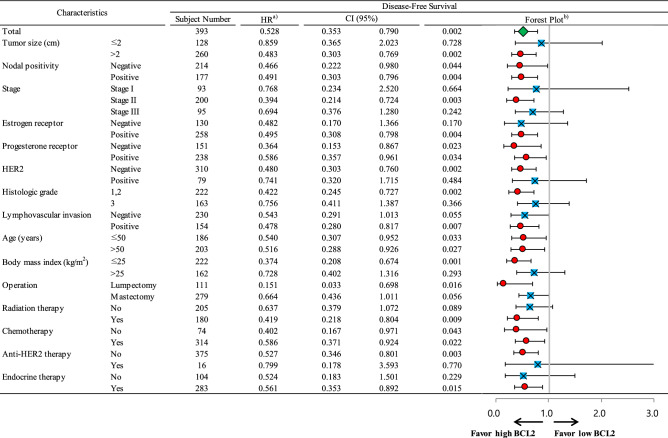
Subgroup analyses by Cox proportional hazards model according to BCL2 status regarding disease-free survival. Abbreviation: CI, confidence interval; HER2, human epidermal growth factor receptor 2; HR, hazard ratio. ^a)^HRs are the relative risks of the high BCL2 group with reference of the low BCL2 group by Cox proportional hazards model. ^b)^In the forest plot, a HR value of less than 1 favors the high BCL2 group against the low BCL2 group. The red circles mean statistical significance and the blue squares mean no statistical significance. The green diamond means the result of total subjects.

**Figure 4:**
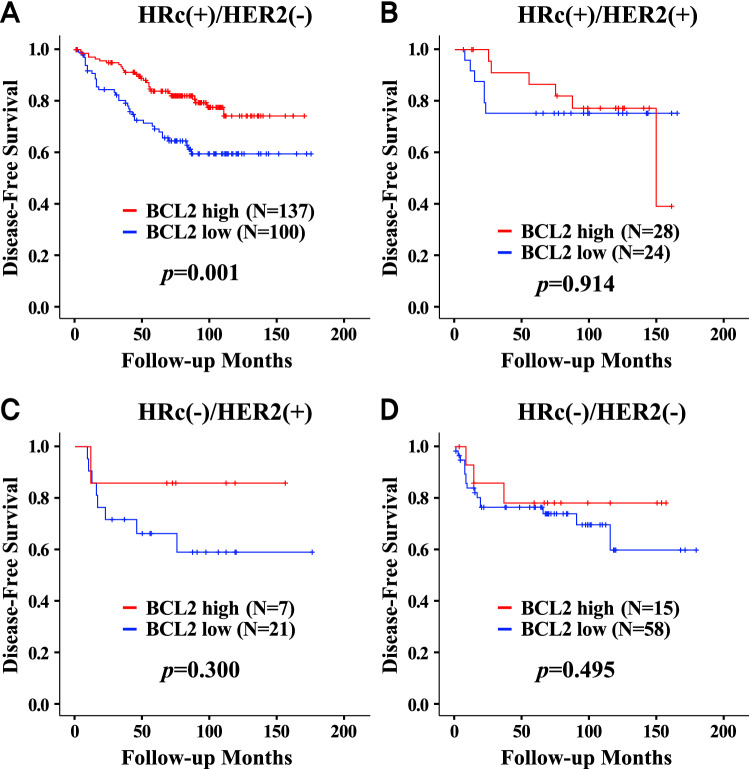
Disease-free survival curve according to BCL2 status in each breast cancer subtype. HRc(+)/HER2(−) (**A**), HRc(+)/HER2(+) (**B**), HRc(−)/HER2(+) (**C**), and HRc(−)/HER2(−) (**D**). Abbreviation: HER2, human epidermal growth factor receptor 2; HRc, hormone receptor; PS, proportion score.

High BCL2 expression was a significant favorable prognostic indicator in all subjects (Fig. 3) regardless of nodal positivity, PR, age group, and chemotherapy. BCL2 expression was also a significant prognostic indicator in subgroups including tumor size > 2 cm, stage II, ER-positive, HER2-negative, histologic grade 1 or 2, positive lymphovascular invasion, and body mass index ≤ 25 kg/m^2^. Additionally, BCL2 expression was a favorable prognostic factor in patients who received lumpectomy, radiation therapy or endocrine therapy, and in patients who did not receive anti-HER2 therapy. Regarding breast cancer subtype, high BCL2 expression was associated with higher disease-free survival in the HRc(+)/HER2(−) subtype only (*p* = 0.001; Fig. 4). BCL2 expression had no association with disease-free survival in the other subtypes including HRc(+)/HER2(+), HRc(−)/HER2(+), and HRc(−)/HER2(−). Of note, prudent interpretation of the negative results in the subtypes of HRc(+)/HER2(+), HRc(−)/HER2(+), and HRc(−)/HER2(−) should be exercised, as the subject numbers of respective subtypes are small.

## Discussion

This study investigated the prognostic roles of BCL1 and BCL2 expression in breast cancer using tissue microarrays from 393 operable primary breast cancer patients. BCL1 expression had no prognostic impact at all but BCL2 expression had significant prognostic effects. The high BCL2 group showed higher disease-free survival compared with the low BCL2 group, especially regarding local recurrence and systemic recurrence. BCL2 expression was a significant independent prognostic factor in terms of disease-free survival.

BCL1 is thought to be a driving oncogene in various solid tumors including breast cancer^1–4,28,29^. *BCL1*, or *cyclin D1*, is amplified in the G1 phase and coordinates the G1 to S phase entry^2^. DNA synthesis of cyclin D1 is complexed with cyclin D-dependent kinases (CDKs), especially with CDK4 and CDK6, and regulates phosphorylation of the retinoblastoma protein, resulting in the release of transcription factors such as E2F-1^30^. These transcription factors then allow cell cycle progression from G1 to S phase. Cyclin D1 and the CDKs are presumably activated in the majority of human tumors and enable cancer cells to enter the cell cycle continuously with a dramatically shortened G1 phase^2^. Several previous studies have proposed the CDK4/6 axis as a rational therapeutic target for cancer treatment^2,31,32^.

As the biological function of BCL1 has been reported to be closely associated with oncogenic activity in human cancer cells, BCL1 was generally predicted to have an adverse effect on the prognosis of breast cancer. Accordingly, some previous studies have reported an adverse prognostic effect of BCL1 in breast cancer patients^8–10^. A study analyzed data from 364 breast cancer patients and reported that high BCL1 expression was associated with worse overall survival in ER-positive cancers^8^. Another study performed meta-analysis using data from 9238 breast cancer patients from 21 different studies and reported that *BCL1* amplification was associated with a higher risk of recurrence and mortality^9^. However, other studies have reported no association between BCL1 and breast cancer prognosis^11,12^. A study that analyzed 1014 breast cancer patients reported that *BCL1* amplification was not associated with significant increases in relapse or death^11^. Another study performed meta-analysis using data from 9189 breast cancer patients from 34 studies and found that high expression of BCL1 was not significantly related to overall survival^12^. Furthermore, other studies have reported that BCL1 is a favorable prognostic indicator^13–15^. A study analyzed data from 179 breast cancer patients and reported that overexpression of BCL1 was associated with higher overall survival^13^. Another study analyzed data from 102 breast cancer patients and reported increased breast cancer-specific survival and increased overall survival in patients with BCL1 expression^14^. As a whole, the prognostic role of BCL1 in breast cancer is largely inconsistent to date.

In this study, although high BCL1 expression was significantly associated with favorable clinicopathologic features such as ER-positive, PR-positive, and low histologic grade, BCL1 expression had no prognostic effect in unselected breast cancers as well as in HRc-positive or HRc-negative breast cancers. While BCL1 has adverse prognostic effects as an oncogene, it has also favorable prognostic effects in breast cancer. BCL1 expression is reported to be associated with well-differentiated breast cancers having favorable clinicopathologic features such as small tumor size, positive ER/PR, and low histologic grade^14,28^. Additionally, BCL1 has been reported to be associated with endocrine therapy resistance in breast cancer^10,33,34^. Favorable effects and adverse effects of BCL1 expression on the breast cancer prognosis could cancel each other, As a whole, BCL1 expression might have no prognostic effect on breast cancer in this study. However, the role of BCL1 as a prognostic indicator in breast cancer is still controversial, and further study is required to conclusively elucidate its effect.

Since BCL2 is a putative oncogene in various malignant tumors, BCL2 expression has been hypothesized to have an adverse prognostic effect in breast cancers. However, most previous studies have reported a favorable prognostic effect of BCL2^16–20^. Callagy et al. analyzed data from 930 breast cancers using tissue microarrays and reported that BCL2 expression is an independent favorable prognostic marker regarding overall survival^16^. They also performed a meta-analysis of 18 studies including 5892 breast cancers and reported the same results^17^. Dawson et al. analyzed 5 studies of 11,212 breast cancers and reported that BCL2 is a powerful favorable prognostic marker irrespective of molecular subtypes or adjuvant therapies received^18^. Hwang et al. also reported that BCL2 is a powerful independent favorable prognostic factor in breast cancer, but the impact of BCL2 was different across breast cancer subtypes^19,20^. Although most previous studies have reported favorable prognostic effects of BCL2, some studies have reported different results. A study analyzed data from 414 breast cancers and reported that BCL2 positivity was not an independent prognosticator in unselected breast cancers^21^. Another study analyzed data from 64 triple-negative breast cancers and reported that high expression of BCL2 is an independent adverse prognostic factor regarding overall survival^22^.

The prognostic influence of BCL2 expression might be different across breast cancer subtypes. Hwang et al. reported that BCL2 expression was a strong prognostic factor in the HRc(+)/HER2(−) subtype^20^, whereas BCL2 expression had only marginal significance in the HRc(+)/HER2(+) subtype, and it was not a significant prognosticator in the HRc(−)/HER2(+) and HRc(−)/HER2(−) subtypes. Dawson et al. reported that BCL2 is a powerful favorable prognostic marker regardless of ER or HER2 status^18^. Another study analyzed data of 2399 breast cancer patients and reported that BCL2 expression is an independent favorable prognostic factor only in the HRc(+)/HER2(−) subtype but not in the other subtypes^35^. In this study, BCL2 expression was a significant prognostic factor only in the HRc(+)/HER2(−) subtype.

The mechanisms through which BCL2 might exert its protective effect in breast cancer are unclear^16,17^. Inhibition of apoptosis is oncogenic, whereas promotion of cell cycle arrest is tumor suppressive. Although BCL2 is known as an anti-apoptotic factor in human malignancies, paradoxical inhibition of solid tumor cell growth by BCL2 has been proposed^36^. BCL2 family members can be both oncogenic and tumor suppressive, and which of the dual functions predominates is lineage-specific and context-dependent^37^. The BCL2 pathway has also been reported to be closely associated with resistance mechanisms to chemotherapy, endocrine therapy, anti-HER2 therapy, and radiation therapy in breast cancer^38–41^. Clinically, BCL2 expression is associated with favorable clinicopathologic features such as small tumor size, negative node, and low histologic grade. BCL2 expression has a strong correlation with HRc-positive and HER2-negative subtypes. These favorable clinicopathologic features could partly explain the superior prognosis of BCL2-positive breast cancers^19,20^. Additionally, BCL2 positivity could be closely related to markers that denote better differentiation of breast cancer cells^16,20,42,43^. High protein expression of BCL2 is observed in luminal subtypes from The Cancer Genome Atlas data^42,43^, and BCL2 expression is dominant in luminal subtypes, especially in luminal A from clinical data^20^. BCL2 expression is down-regulated in non-luminal subtypes such as HER2 and triple-negative subtypes which are related to worse prognoses^43^.

This study investigated the prognostic impacts of BCL1 and BCL2 expression in breast cancer by utilizing tissue microarray data of breast cancer patients. While detailed clinicopathologic information and long-term follow-up duration are the strengths of our study, it is important to denote the limitations. First, the number of subjects is relatively small, which might lessen the statistical power, especially in subgroup analyses. In particular, the negative results in the subtypes of HRc(+)/HER2(+), HRc(−)/HER2(+), and HRc(−)/HER2(−) might be rather inconclusive which need to be validated using larger number of subjects. Second, the cut-off values of the BCL1 and BCL2 expression are arbitrary because there are no standardized methods. Further studies are needed to overcome these limitations and validate the prognostic roles of BCL1 and BCL2.

In conclusion, BCL1 expression had no prognostic impact on disease-free survival in breast cancer, whereas BCL2 expression was a significant independent prognostic factor in terms of disease-free survival. The high BCL2 group displayed superior disease-free survival compared with the low BCL2 group, especially regarding local recurrence and systemic recurrence. The favorable prognostic effect of BCL2 expression was detected only in the HRc(+)/HER2(−) subtype, but not in the other subtypes including HRc(+)/HER2(+), HRc(−)/HER2(+), and HRc(−)/HER2(−). Favorable clinicopathologic features and strong association with ER/PR status could partly explain the superior prognosis of the high BCL2 group. BCL2 expression could be utilized to predict prognosis of operable breast cancer patients in clinical settings. As low BCL2 expression is associated with unfavorable clinical outcomes, more active adjuvant treatment might be recommended for breast cancer patients with low BCL2 expression. Further studies are required to validate the usefulness of BCL2 expression as a prognostic marker in breast cancer.

## Supplementary Information

Supplementary Information 1.

## Abbreviations

CDK: Cyclin D-dependent kinase
CI: Confidence interval
ER: Estrogen receptor
HER2: Human epidermal growth factor receptor 2
HR: Hazard ratio
HRc: Hormone receptor
PR: Progesterone receptor
