# Supplementary material for: Prognostic influences of BCL1 and BCL2 expression on disease-free survival in breast cancer
Source: Sci Rep. 2021 Jun 7;11:11942. doi: 10.1038/s41598-021-90506-x (PMC8184896; doi:10.1038/s41598-021-90506-x)
Supplement: Supplementary file 1 — Supplementary Information 1. [file 41598_2021_90506_MOESM1_ESM.pdf]

[Original Research]

Prognostic Influences of BCL1 and BCL2 Expression on Disease-Free Survival in Breast Cancer

Ki-Tae Hwang[1,*,†], Young A Kim[2,*], Jongjin Kim[1], Hyeon Jeong Oh[3], Jeong Hwan Park[2], In Sil Choi[4], Jin Hyun Park[4], Sohee Oh[5], Ajung Chu[6], Jong yoon Lee[6] & Kyu Ri Hwang[7]

[1]Department of Surgery, Seoul Metropolitan Government Seoul National University Boramae Medical Center, Seoul, Republic of Korea
[2]Department of Pathology, Seoul Metropolitan Government Seoul National University Boramae Medical Center, Seoul, Republic of Korea
[3]Department of Pathology, National cancer center, Goyang-si, Gyeonggi-do, Republic of Korea
[4]Department of Internal Medicine, Seoul Metropolitan Government Seoul National University Boramae Medical Center, Seoul, Republic of Korea
[5]Medical Research Collaborating Center, Seoul Metropolitan Government Seoul National University Boramae Medical Center, Seoul, Republic of Korea
[6]Department of Radiology, Seoul Metropolitan Government Seoul National University Boramae Medical Center, Seoul, Republic of Korea
[7]Department of Obstetrics & Gynecology, Seoul Metropolitan Government Seoul National University Boramae Medical Center, Seoul, Republic of Korea

Email address:
Ki-Tae Hwang, MD, PhD, kiterius@snu.ac.kr, 0000-0001-6597-3119 (ORCID)
Young A Kim, MD, PhD, youngakim@gmail.com, 0000-0002-8253-2881 (ORCID)
Jongjin Kim, MD, michael5@hanmail.net, 0000-0001-5234-7856 (ORCID)
Hyeon Jeong Oh, MD, PhD, jumper6074@gmail.com, 0000-0002-9998-3988 (ORCID)
Jeong Hwan Park, MD, PhD, hopemd@hanmail.net, 0000-0003-4522-9928 (ORCID)
In Sil Choi, MD, PhD, hmoischoi@hanmail.net, 0000-0002-8494-584x (ORCID)
Jin Hyun Park, MD, PhD, jinhyunpak@gmail.com, 0000-0003-0178-4139 (ORCID)
Sohee Oh, PhD, oh.sohee@gmail.com, 0000-0002-3010-448X (ORCID)
Ajung Chu, MD, ajstyle83@gmail.com, 0000-0003-2018-6706 (ORCID)
Jong yoon Lee, MD, leeage@naver.com, 0000-0002-0070-0862 (ORCID)
Kyu Ri Hwang, MD, PhD, orangemd@snu.ac.kr, 0000-0001-6845-1260 (ORCID)

[*]**First authors:** Ki-Tae Hwang and Young A Kim contributed equally to this work.
[†]**Correspondence:** Ki-Tae Hwang, MD, PhD
Department of Surgery, Seoul Metropolitan Government Seoul National University Boramae Medical Center
39, Boramae-Gil, Dongjak-gu, Seoul, 156-707, Republic of Korea
Tel: 82-2-870-2275; Fax: 82-2-831-2826; E-mail: kiterius@snu.ac.kr

Running title: BCL1, BCL2 and Breast Cancer Prognosis

Conflict of interest: The authors declare that no actual or potential conflict of interest exists. The institutional review boards approved this study (Seoul Metropolitan Government Seoul National University Boramae Medical Center, 16-2016-82).

Grant sponsor: This research was supported by a multidisciplinary research grant-in-aid from the Seoul Metropolitan Government Seoul National University Boramae Medical Center (02-2018-4) and by Research Program 2019 funded by Seoul National University College of Medicine Research Foundation (800-20190513).

Abbreviations: CDK, cyclin D-dependent kinase; CI, confidence interval; ER, estrogen receptor; HER2, human epidermal growth factor receptor 2; HR, hazard ratio; HRc, hormone receptor; PR, progesterone receptor.

Table S1. Detailed disease-free survival rates according to BCL2 status.

Follow-up duration (month)	Disease-Free Survival								
	BCL2 low			BCL2 high			Total		
	Subject No	Event No[a)]	Rate	Subject No	Event No[a)]	Rate	Subject No	Event No[a)]	Rate
60	205	52	73.2%	188	22	87.7%	393	74	80.1%
120	127	65	63.8%	144	35	78.0%	271	100	70.7%
180	50	66	61.6%	50	37	72.5%	100	103	67.0%
Last follow-up[b)]	7	66	61.6%	7	37	72.5%	14	103	67.0%

[a)]Cumulative number of events.

[b)]Last follow-up durations were 216 months, 205 months, and 216 months for the low BCL2 group, high BCL2 group and total group, respectively.

Figure S1. Immunohistochemical staining of BCL1 and BCL2 in normal breast tissues and breast cancer tissues (×200). BCL1 staining is not detected in normal breast tissues (A). A case with negative staining of BCL1 in the breast cancer tissue (B), and a case with positive staining of BCL1 in the breast cancer tissue (C). BCL2 staining is detected in normal breast tissues (D). A case with negative staining of BCL2 in the breast cancer tissue (E), and a case with positive staining of BCL2 in the breast cancer tissue (F). Scale bar (bottom right), 100 μm.

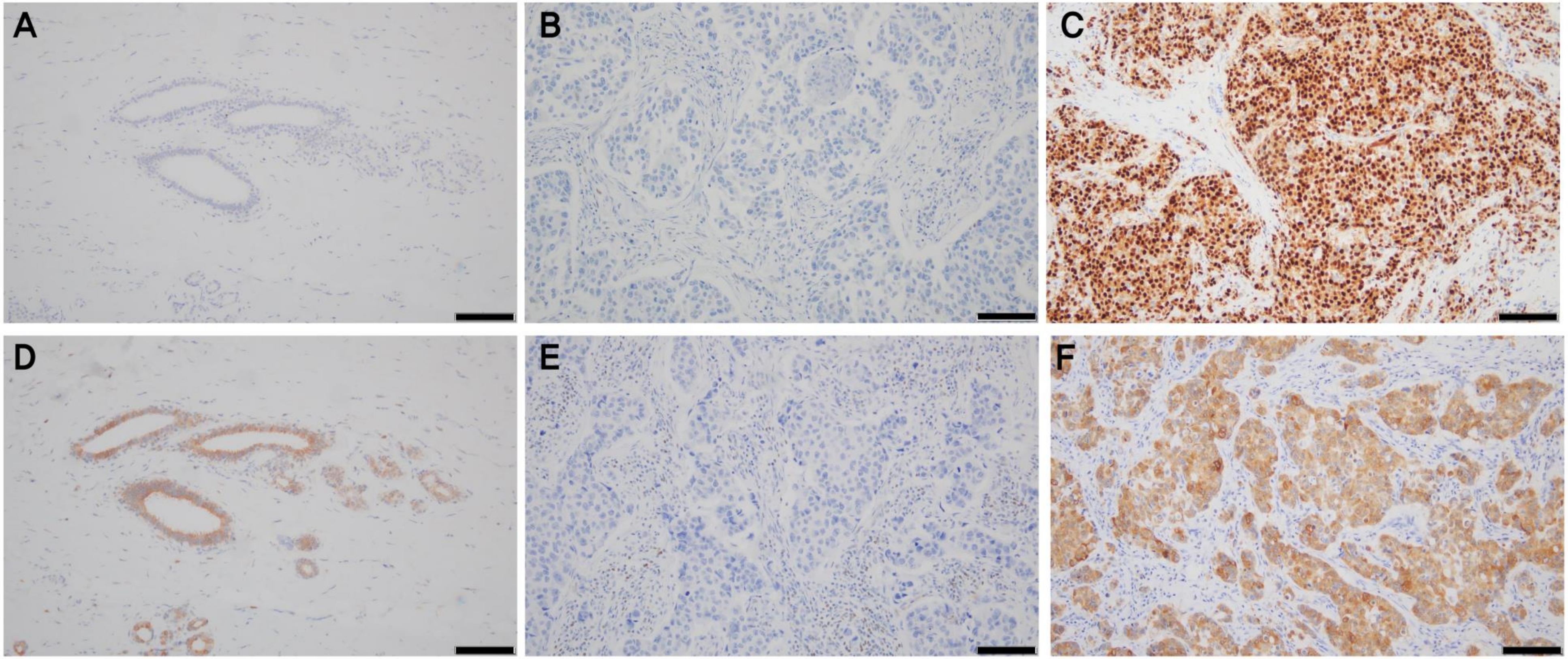

Figure S2. Immunohistochemical staining results of BCL1 (×200). Proportion score of less than 10% (A), and more than 10% (B). Intensity score of 0 (C), 1 (D), 2 (E), and 3 (F). Scale bar (bottom right), 100 μm.

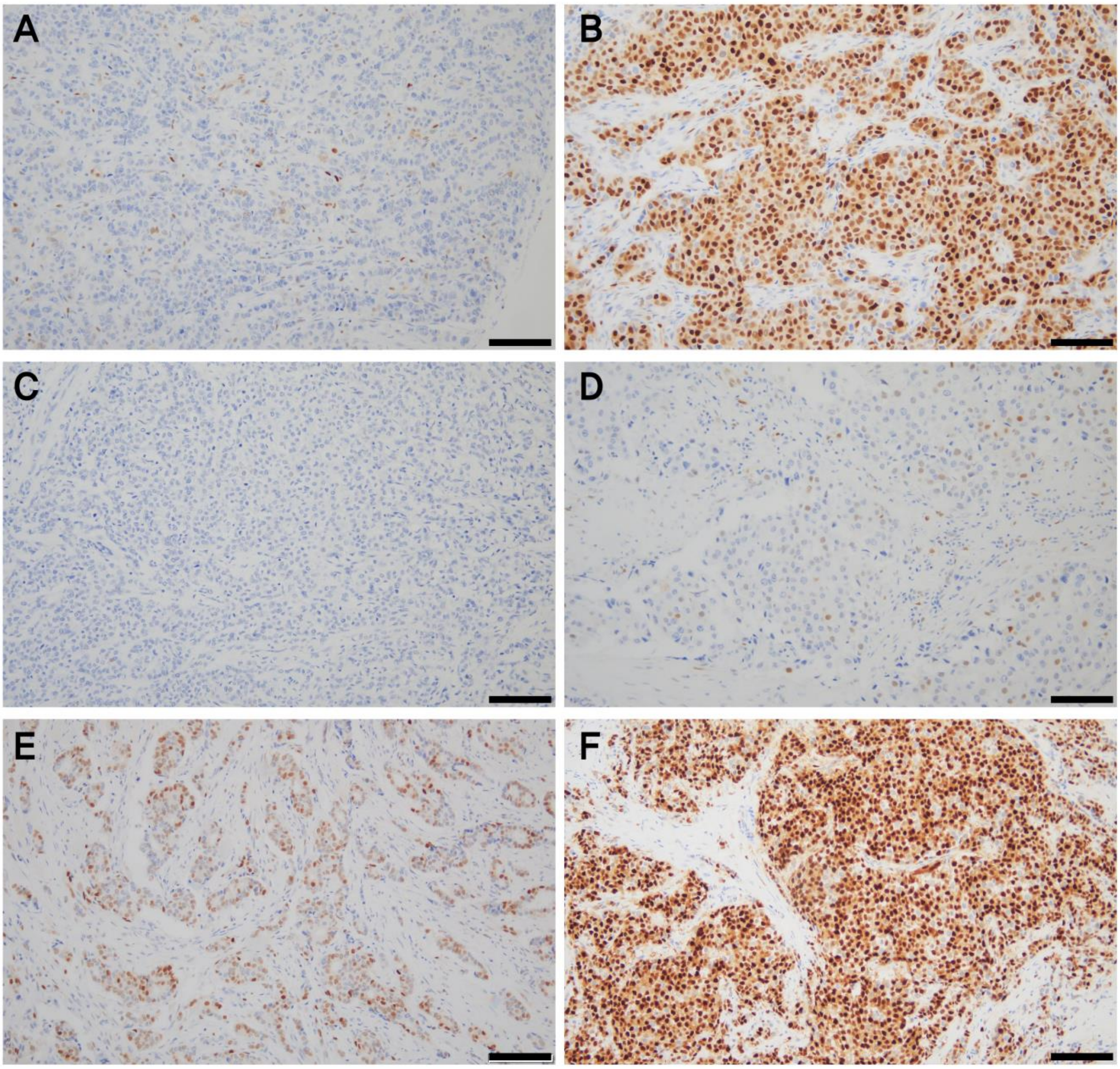

Figure S3. Immunohistochemical staining results of BCL2 (×200). Proportion score of 10% (A), and more than 10% (B). Intensity score of 0 (C), 1 (D), 2 (E), and 3 (F). Scale bar (bottom right), 100 μm.

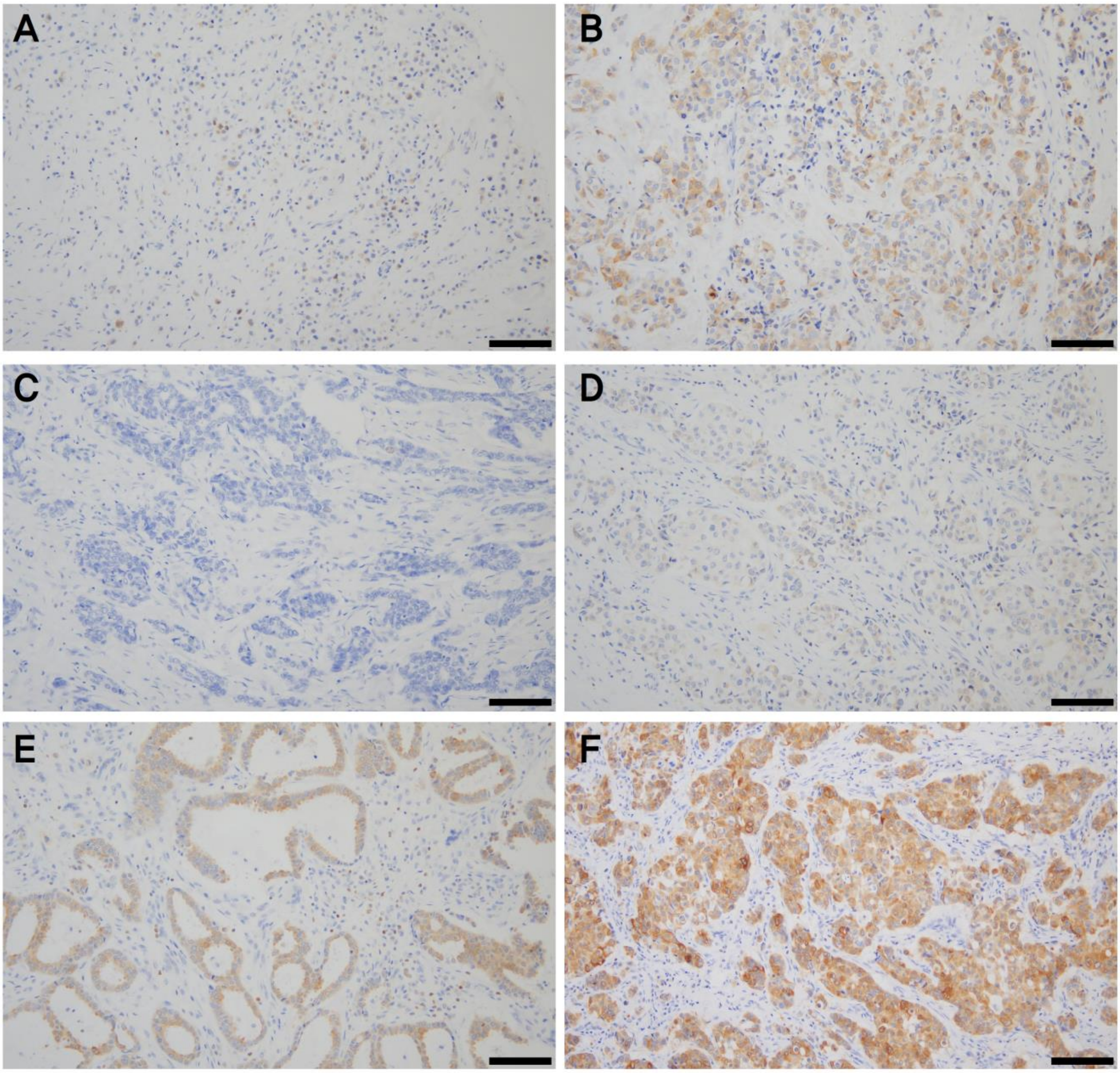

Figure S4. Disease-free survival curves according to BCL1 status with each cut-off value of Allred score. The high BCL1 group was defined as Allred score >2 (A), Allred score >3 (B), Allred score >4 (C), Allred score >5 (D), Allred score >6 (E), and Allred score >7 (F), respectively.

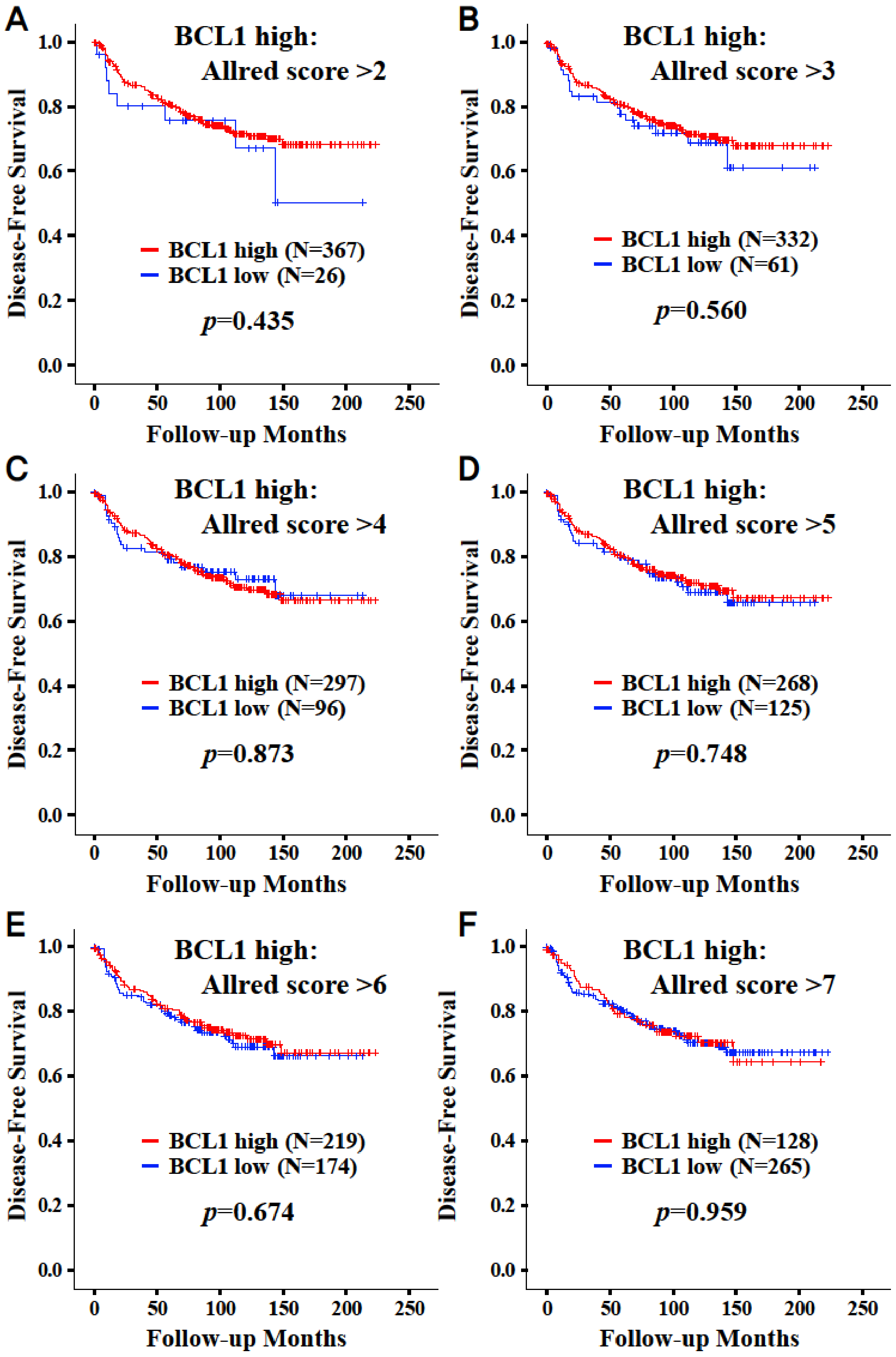

Figure S5. Disease-free survival curves according to BCL2 status with each cut-off value of Allred score. The high BCL2 group was defined as Allred score >2 (A), Allred score >3 (B), Allred score >4 (C), Allred score >5 (D), Allred score >6 (E), and Allred score >7 (F), respectively.

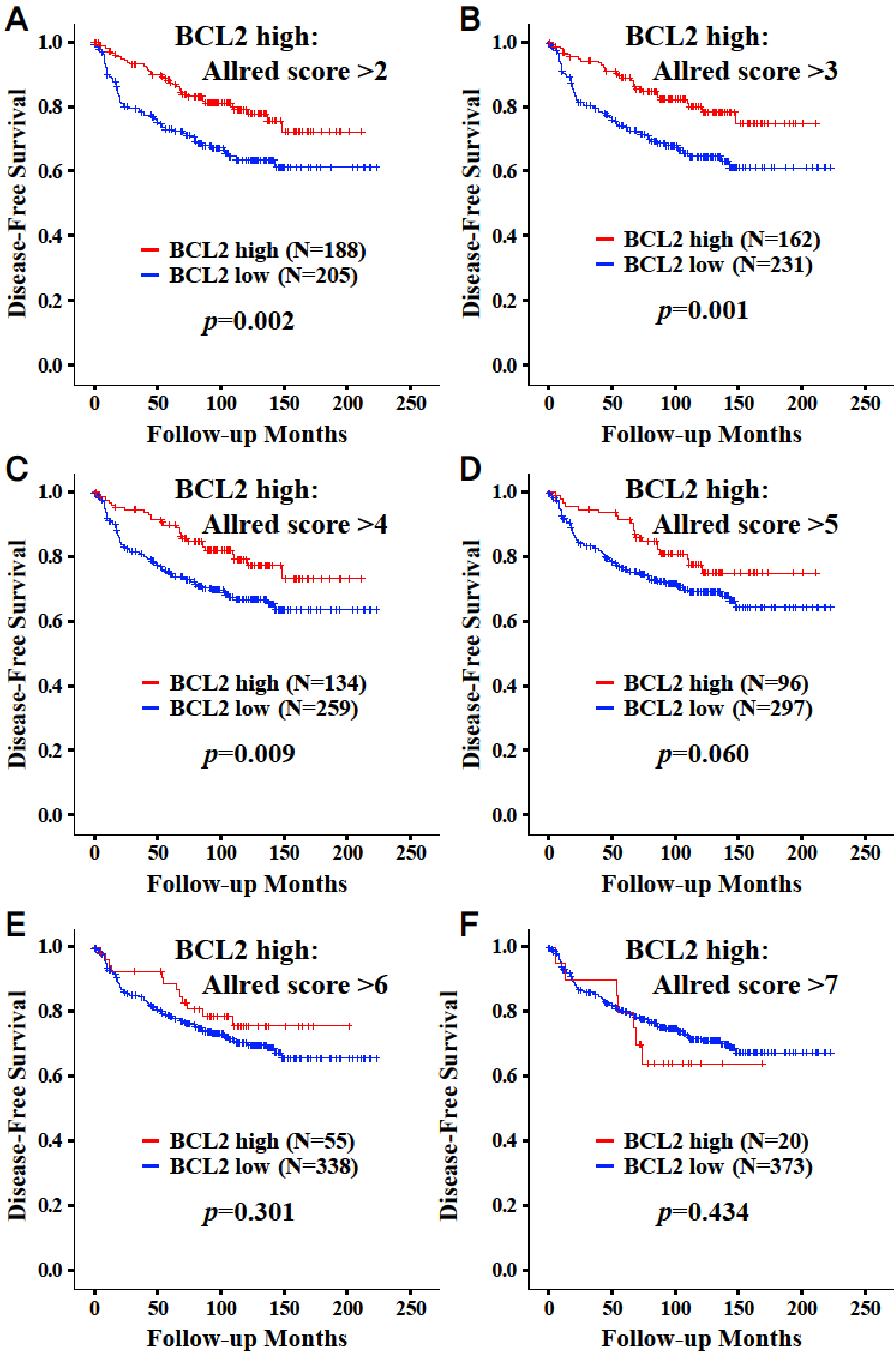

Figure S6. Disease-free survival curves according to BCL1 and BCL2 statuses. Survival curves according to BCL1 status defined by intensity score (A) and proportion score (B). Survival curves according to BCL2 status defined by intensity score (C) and proportion score (D).

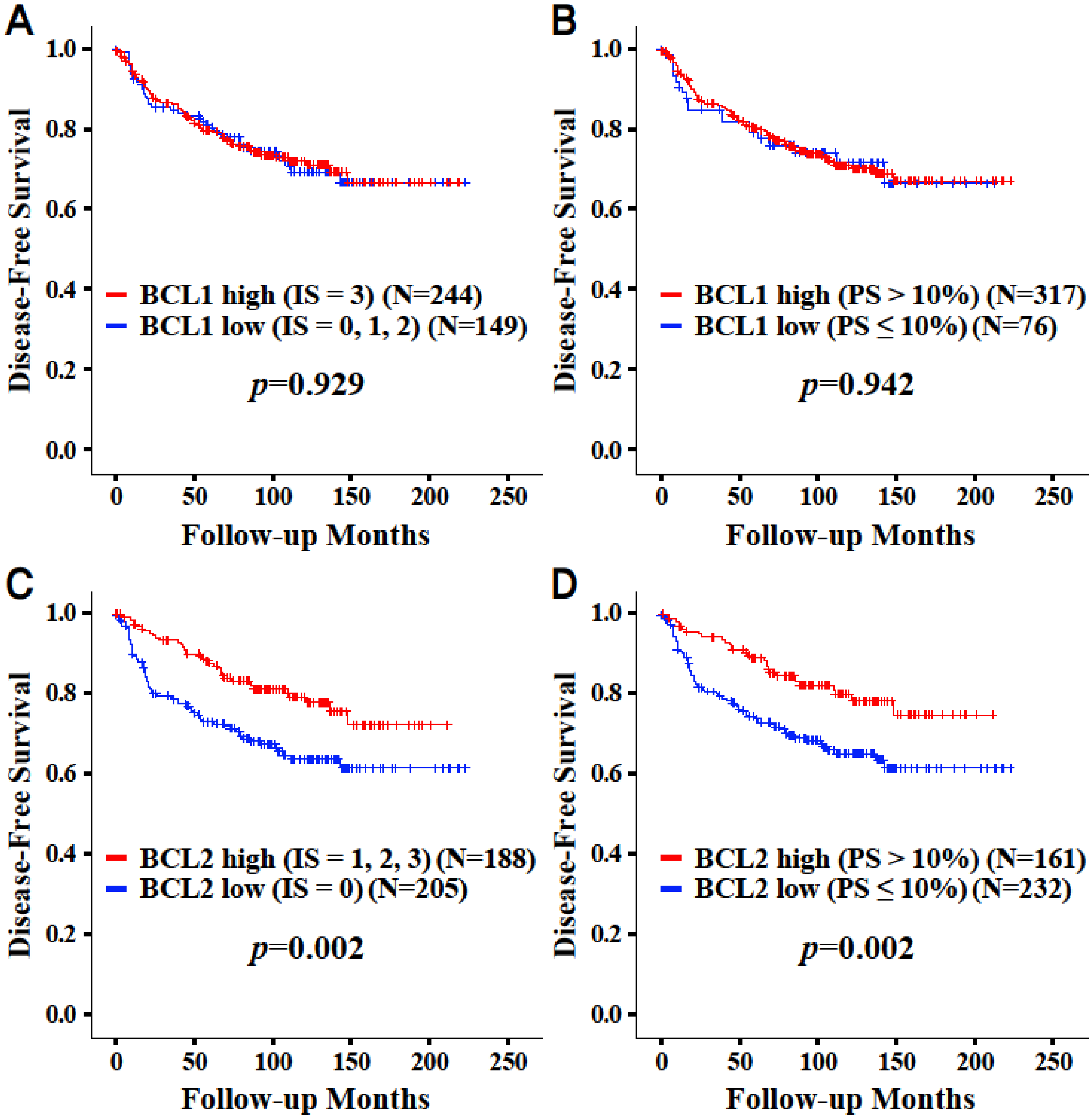

Abbreviation: IS, intensity score; PS, proportion score.

Figure S7. Disease-free survival curves according to BCL2 status. Survival curves according to BCL2 status in HRc-negative (A) and HRc-positive breast cancers (B).

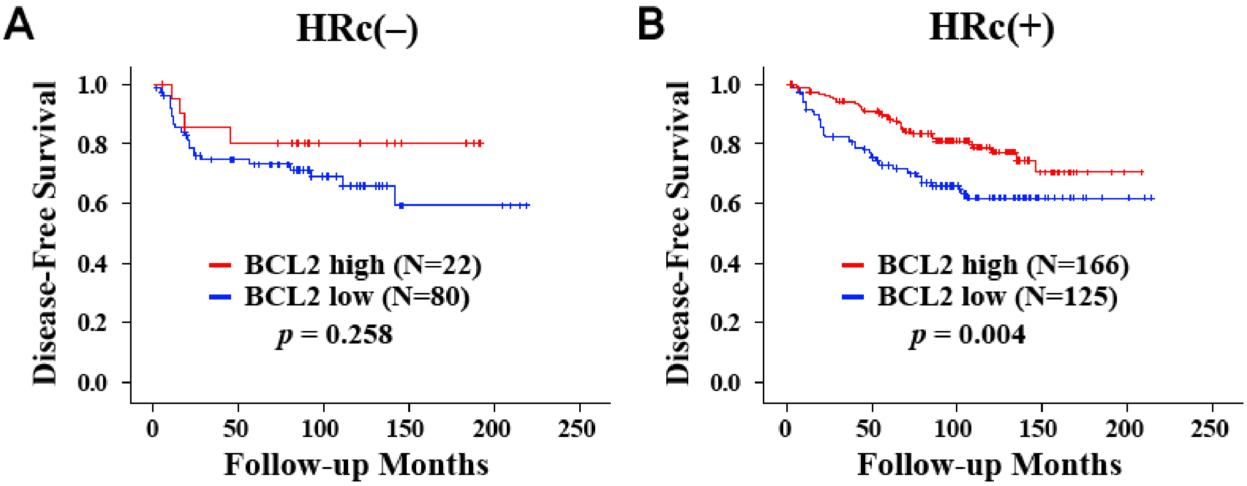

Abbreviation: HRc, hormone receptor.
